# Empowering pharmacists in asthma management through interactive SMS (EmPhAsIS): study protocol for a randomized controlled trial

**DOI:** 10.1186/1745-6215-15-488

**Published:** 2014-12-13

**Authors:** Mary A De Vera, Mohsen Sadatsafavi, Nicole W Tsao, Larry D Lynd, Richard Lester, Louise Gastonguay, Jessica Galo, J Mark FitzGerald, Penelope Brasher, Carlo A Marra

**Affiliations:** Faculty of Pharmaceutical Sciences, University of British Columbia, 2405 Wesbrook Mall, Vancouver, V6T 1Z3 Canada; Faculty of Medicine, University of British Columbia, 317 - 2194 Health Sciences Mall, Vancouver, BC V6T 1Z3 Canada; Centre for Clinical Epidemiology and Evaluation, 7th Floor, 828 West 10th Avenue Research Pavilion, Vancouver, V5Z 1M9 Canada; School of Pharmacy, Memorial University of Newfoundland, Health Sciences Centre 300 Prince Philip Drive, St John’s, A1B 3V6 Canada

**Keywords:** Asthma, Medication adherence, Inhaled corticosteroids, Cluster randomized controlled trial, Community pharmacy, Pharmacy practice research

## Abstract

**Background:**

Medication regimens for asthma are particularly vulnerable to adherence problems because of the requirement for long-term use and periods of symptom remission experienced by patients. Pharmacists are suited to impact medication adherence given their training, skills, and frequent contact with patients. The Empowering pharmacists in asthma management through interactive SMS (EmPhAsIS) trial involves an intervention leveraging mobile health (mHealth) technology to support community pharmacy practice with the hypothesis of improved medication adherence in asthma.

**Methods/Design:**

This study is a pragmatic pharmacy-based, cluster, randomized controlled trial with 12 months of intervention delivery and follow-up. Pharmacies (the clusters) will be randomized at a 1:1 ratio to provide intervention or usual care. The EmPhAsIS intervention consists of patient asthma education, short message service (SMS)-based monthly assessment of adherence, and follow-up of non-adherent individuals by community pharmacists. There are no inclusion or exclusion criteria for pharmacies. Patients are eligible if they: are 14 years of age or older, fill a prescription for inhaled corticosteroid (either monotherapy or in a combination inhaler with long-acting beta-agonists), have been diagnosed with asthma, possess a mobile phone with SMS capabilities, and have no communication difficulties such as inability to communicate in English, or significant impairment in vision, hearing, or speech. The primary outcome is adherence to inhaled corticosteroids ascertained by the medication possession ratio, the ratio of the days of medication supplied to days in a given time interval. This study will also evaluate secondary outcomes including: asthma control, asthma-related quality of life, asthma-related hospital admissions, and use of reliever medications during the follow-up period. A nested economic evaluation using a probabilistic decision-analytic model will be used to perform a cost-effectiveness analysis from the societal perspective of the intervention compared with usual care over a 10-year time horizon.

**Discussion:**

Considering the prevalence of asthma, the extent of the non-adherence problem in this disease, and the availability of effective treatments, there is a tremendous potential to reduce the burden of asthma through improving adherence. This is the first study of an intervention based on mobile communication technology involving community pharmacists in asthma management.

**Trial registration:**

ClinicalTrials.gov identifier: NCT02170883; date of registration: 19 June 2014.

## Background

Asthma is a chronic inflammatory disease of the airways which is characterized by recurrent, but reversible, episodes of shortness of breath, chest tightness, coughing, and wheezing [1]. The global prevalence of asthma is estimated at 300 million [2, 3] and the condition affects individuals of all ages [4]. In 2010, more than 2.4 million Canadians aged 12 years or older reported that they have been diagnosed as having asthma [5]. The ultimate goal of asthma management is to bring the disease under clinical control [6]. Compared to controlled asthma, uncontrolled asthma is associated with increased medical costs, reduced quality of life, and loss of productivity [7].

Pharmacotherapy remains the cornerstone of asthma management. All modern guidelines recommend daily use of controller medications regardless of the level of asthma impairment (with the exception of very mild and intermittent asthma) [6, 8]. Currently, inhaled corticosteroids (ICS) are widely accepted as the primary controller therapy for asthma [6]. Yet despite recommendations, there is a disappointingly low level of adherence to asthma controller therapies [9]. The average rate of adherence to inhaled corticosteroids for asthma is reported to be between 22 and 63% [10–16]. Medication regimens for asthma are particularly vulnerable to adherence problems because of the requirement for long-term use and the long periods of symptom remission experienced by patients [17]. The relationship between adherence to controller therapies and short- and long-term adverse asthma-related outcomes is well established [18, 19]. Landmark studies in the 1990s showed that regular treatment with ICS is associated with up to an 80% reduction in the risk of death, as well as severe asthma exacerbations requiring hospitalization [20–23].

Pharmacists are ideally suited to impact medication adherence given their training, skills, and frequent contact with patients; up to eight times more often than doctors [24]. A few studies have evaluated community-pharmacist-based interventions in the management of asthma. A Spanish cluster randomized controlled trial (RCT), in which patients in the intervention group scheduled three to six visits to their pharmacist over six months of follow-up, reported that adherence was improved by 40.3% in the intervention group compared with the control group [25]. Similarly, an Australian cluster RCT with 50 pharmacies (396 patients), involving an ongoing cycle of assessment, goal setting, monitoring, and review by pharmacists, showed that compared with usual care, the intervention resulted in improved adherence to controller medication (odds ratio for adherence: 1.89) [26]. A cluster RCT of community pharmacists in British Columbia (BC) also showed promising results of an ‘enhanced care protocol’, with pharmacists responsible for assessing a patient’s readiness to change, tailoring asthma education to that readiness, monitoring compliance, and collaborating with physicians to achieve asthma prescribing guidelines [27].

Mobile phones are globally the most pervasive and accessible form of two-way communication technology [28]. The use of mobile phones in public health practice (mobile health, mHealth) [29] offers opportunities to enhance patient care and impact medication adherence across many diseases. Previous studies have considered text messages to be a behavior change communication modality [30]; a process of any intervention with individuals to develop communication strategies to promote positive behaviors [31]. In a multisite randomized clinical trial of HIV-infected adults initiating antiretroviral therapy (ART) in three clinics in Kenya, patients were randomized at a 1:1 ratio to receive weekly short message service (SMS) intervention coupled with clinic nurse follow-up, or standard care. The primary outcome (adherence to ART) was reported in 168 of 273 patients receiving the SMS intervention compared with 132 of 265 in the control group (relative risk (RR) for non-adherence: 0.81; 95% CI: 0.69 to 0.94; *P* = 0.006) [32].

To overcome the epidemic of low adherence, our healthcare system requires innovative models of care that fully harness the knowledge, skills, availability, and enthusiasm of healthcare providers, and facilitate communication across the chain of healthcare delivery [33]. In a critical review of the literature on adherence interventions in asthma, Bender *et al*. concluded that research is encouraged into innovative interventions that are brief, easily implemented, and can be tailored to individual patients and diverse clinical settings [33]. Previous studies have evaluated communication technologies in asthma care [34, 35], but none of these studies have involved pharmacists. On the other hand, pharmacist-based studies on improving the management of asthma have hitherto used interventions whose real-world feasibility and cost-effectiveness have not been evaluated. Altogether, we perceive a role for mHealth to support community pharmacy practice and provide a means for an accessible and cost-effective method of communication between asthma patients, with the ultimate goal of facilitating patient engagement in their disease management, thereby improving adherence and outcomes [35].

## Methods/Design

### Study design

This trial, known as ‘Empowering pharmacists in asthma management through interactive SMS’ (EmPhAsIS), will be implemented from 2015 to 2018 in BC, Canada. It is a pragmatic, cluster RCT trial of a community-pharmacist-initiated, mHealth-supported, adherence intervention (EmPhAsIS intervention) for asthma, with 12 months of participant (pharmacies and patients) recruitment and 12 months of follow-up over which the intervention will also be delivered. Community pharmacies in the province of BC define the clusters based on three reasons. First, patient-level randomization would require each study pharmacist to provide either the intervention or usual care to different patients of the same pharmacy and can lead to contamination. Second, pharmacist-level randomization would require the pharmacist to be both the participant recruiters and randomization units which could lead to selection bias concerns (for example, pharmacists randomized to the intervention group preferentially recruiting patients whom they know are at higher risk of medication non-adherence). Third, given that most pharmacists in community pharmacies are scheduled on a shift-work basis and provide overlapping care to all patients of the pharmacy, the risk of contamination cannot be practically controlled in this environment. To eliminate such randomization problems, it was therefore pragmatically decided to randomize clusters at an organizational level and make these services available on a per pharmacy basis. This trial has been approved by the University of British Columbia Clinical Research Ethics Board (approval number: H14-01451).

### Objectives

Our primary objective is to compare adherence to ICS between asthma patients attending pharmacies assigned to provide the EmPhAsIS intervention to usual care. Our secondary objective is to evaluate the impact of the EmPhAsIS intervention on asthma control, asthma-related quality of life, asthma-related hospital admissions, and use of reliever medications during the follow-up period. Our third objective is to evaluate the cost-effectiveness of the EmPhAsIS intervention from a societal perspective over a 10-year time horizon.

### Participants

#### Pharmacies

Pharmacies will be recruited from an existing database of community pharmacies we have partnered with, of which pharmacists have indicated interest in collaborating with us. Pharmacies (the clusters) will be randomized in a 1:1 ratio to intervention or usual care. All recruited pharmacies will receive pamphlets that will describe the study protocol and the Global Initiative for Asthma (GINA)’s Pocket Guide for Asthma [36]. All pharmacists will attend an online workshop (webinar) during which they will be trained on the study protocol and provided education on asthma and medication adherence as part of the study. Pharmacists will also attend separate webinars (intervention webinar and usual care webinar) and will receive written study manuals specific to whether they have been assigned to the EmPhAsIS intervention or usual care group. There are no inclusion or exclusion criteria for pharmacies.

#### Patients

Patients eligible for this study are individuals who fill a (incident or prevalent) prescription for ICS (either monotherapy or in a combination inhaler with long-acting beta-agonists) who answer affirmatively to the question ‘Have you ever been diagnosed by a doctor as having asthma?’. In line with the principles of pragmatic trials, our inclusion criteria are not overly restrictive so that the study sample remains representative of the target population. These inclusion criteria are similar to recent pragmatic trials in asthma [37] and are the following: 1) aged 14 years or older; 2) possessing a cellphone with the ability to send and receive text messages; 3) residing in BC, and planning to reside in BC for the next 12 months; 4) having been registered with the medical services plan (MSP, the provincial insurer of medically-required services) in the past 12 months, and planning to remain registered for the next 12 months; 5) not participating in another interventional study; 6) no communication difficulties, such as inability to communicate in English, or significant impairment in vision, hearing, or speech; and 7) consenting to participate in the study. Written informed consent will obtained from eligible patients before enrollment into the study.

We will utilize recruitment strategies used in our prior pharmacy practice studies [38–40], including posters and shelf-talkers. We will also advertise the study through various communication channels available to our research team, including the study website and social media. We will also implement strategies for targeting recruitment, including working closely with participating pharmacies to establish recruitment targets that are appropriate for the community they service. Furthermore, we will also implement regular monitoring of recruitment, including communication (for example, site visits, telephone calls, and/or emails) to discuss challenges and progress as well as offer ongoing support.

### Randomization

Assignment of pharmacies to provide the EmPhAsIS intervention or usual care will be done in a 1:1 ratio with randomly permuted blocks in sizes of four and six. A computer-generated list will be used to ensure balance in the distribution of intervention and usual care groups at any point in the trial while minimizing the risk that research staff may be able to predict group assignment. Clusters will be allocated to intervention or usual care group by one designated research staff member using the computer-generated list. It will not be possible for participating pharmacies to be blinded to which group they are assigned. However, while the trial will be managed by a coordinator who will also be unblinded, team members responsible for data collection (an interviewer who will collect patient-reported data at 0, 6, and 12 months) and analyses (statistical analyst) will be blinded to group allocation.

### Study groups

#### Intervention (EmPhAsIS) group

Patients attending pharmacies assigned to the intervention group will receive the EmPhAsIS intervention which consists of three main pillars: a) patient education, b) SMS-based monthly assessment of adherence to controller therapies, and c) follow-up of non-adherent individuals by community pharmacists.

**Figure 1:**
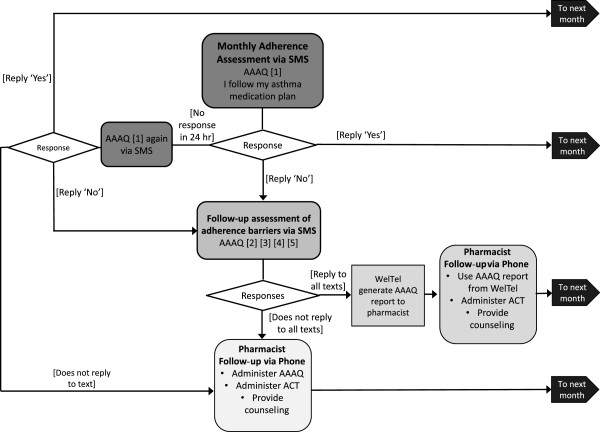
**Schematic of transmission of short message service (SMS) assessment of adherence (pillar two) and follow-up of non-adherent patients by community pharmacist (pillar three) of the EmPhAsIS intervention.** AAAQ: Adult Asthma Adherence Questionnaire; ACT: Asthma Control Test.

- Patient education: the education component will involve the pharmacist discussing patients’ individual treatments, information on the chronic, episodic nature of asthma and the importance of continuous controller therapy, with emphasis on medication adherence.
- SMS-based monthly assessment of adherence to controller therapies: The principal component of the intervention is monthly text messages, delivered over the 12 month follow-up period, by which patients are asked to provide their level of agreement with the statement ‘I follow my asthma medication plan’. Responses range on a Likert scale (1: Agreeing completely, 2: Agreeing mostly, 3: Agreeing somewhat, 4: Disagreeing somewhat, 5: Disagreeing mostly, and 6: Disagreeing completely). Individuals will be asked to respond by typing back a number (between one and six) corresponding to the response items. This question and the set of responses are the first item in the Adult Asthma Adherence Questionnaire™ (AAAQ) [41]. The AAAQ is a five-item questionnaire developed specifically as a screening diagnostic tool for adherence by care providers, as well as for identifying potential adherence barriers [42]. The first question is a general adherence monitoring question, and questions two to five determine specific barriers to adherence among individuals with low adherence (forgetting, no need, adverse effects, and costs). The AAAQ has been shown to have a high degree of construct validity and has proven to be a strong predictor of adherence, as measured through administrative health data [42]. There are alternative adherence questionnaires [43, 44], but they do not have the predictive power of the AAAQ [44], or consist of too many questions and thus are cumbersome to administer [43]. In addition, the AAAQ is particularly suited to the EmPhAsIS intervention given the separation of the adherence-monitoring question (question one) from those determining specific barriers to adherence (questions two to five). The transmission and receipt of text messages will be centralized and automated using the University of British Columbia (UBC) designed WelTel system. WelTel is an SMS support and engagement platform that will be administered at our research centre and accessed online by pharmacists through the study website. Centralizing text message transmissions minimizes the burden on pharmacists who will not have to send messages themselves, and reduces the delays and potential errors in the evaluation of responses.
- Follow-up of non-adherent individuals by community pharmacists: If the response to item one of AAAQ, received through SMS, is anything other than one (Agreeing completely), then this would indicate an adherence problem and the individual will be asked the rest of the adherence-barriers questions of the AAAQ (questions two to five) via SMS. Based on the responses, WelTel will generate an AAAQ adherence report that identifies potential adherence barrier(s) (such as cost or fear of side effects) to help facilitate and guide the pharmacist’s follow-up telephone call with the patient. A pharmacist will then follow-up with the individual within the next 24 hours. During the telephone follow-up, the pharmacist will administer the Asthma Control Test (ACT, a five-item validated and widely used questionnaire assessing asthma control) [41]. In separate training provided to pharmacists in the EmPhAsIS intervention group, webinars will provide step-by-step instruction on use of the WelTel platform as well as administration of the ACT over the telephone. Patient responses to the ACT items will be entered into a survey system that will automatically score the questionnaire in order to facilitate this step for the pharmacist. Based on the individual’s response to the ACT, the outcome of this phone interview may be counselling and education to address adherence problems, or a referral to the patient’s physician (if the individual receives a score of 19 or lower in the ACT, which may indicate the presence of uncontrolled asthma). For patients where the adherence problem is unintentional, the pharmacist will provide phone-based counselling and support, for example by clarifying instructions, offering suggestions on incorporating medication taking with the patient’s daily routine, and offering adherence tools such as calendars. For patients where the adherence problem is intentional, the pharmacist can provide education on the risks and benefits of treatment and non-treatment, evaluate adverse effects, and consider medication coverage options (or the use of alternative formulations). Figure 1 illustrates SMS transmission for pillars two and three of the EmPhAsIS intervention along with scenarios for instances of non-responses to the monthly SMS message. Pharmacists will log telephone calls, including response and non-responses, and in instances of non-responses, at least two follow-up attempts will be made.

#### Usual care (control) group

Patients attending pharmacies assigned to the control group will receive usual care. However, it is imperative that the study can distinguish between the impact of the intervention itself and the training that pharmacists and patients receive upon recruitment into the study. Indeed, previous adherence-intervention studies have been criticized because the individuals in the intervention group were more likely to receive an evidence-based education [33]. As such, patients in the control group will receive the patient education component of the EmPhAsIS intervention (pillar one).

### Data collection and study follow-up

**Figure 2:**
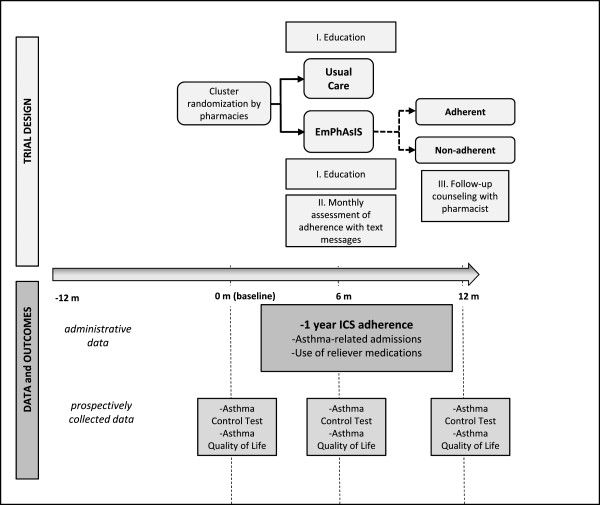
**EmPhAsIS trial design and schematic of data collection and outcomes.** ICS: inhaled corticosteroids.

The design of the trial and the schematic of data collection and outcomes are illustrated in Figure 2. Pharmacists will screen for eligibility, obtain informed consent, and enroll patients at their pharmacy. Upon patient enrollment, pharmacists will securely fax the individual’s contact information to the study research coordinator. Prospective data will be collected by telephone interview by an interviewer at set time points as follows: 1) sociodemographic characteristics, such as age, education, income, BC provincial health number (baseline); 2) Asthma Control Test and Asthma Quality of Life Questionnaire (AQLQ) (baseline, 6 months, and 12 months); and 3) changes in the enrollment of the individual with the provincial healthcare system, changes in third-party insurance, as well as the frequency of receiving inhaler medications through samples. In addition to prospectively collected outcome data, we will also obtain data for our study sample through BC PharmaNet (http://www.health.gov.bc.ca/pharmacare/pharmanet/netindex.html) [45] and Population Data BC (http://www.popdata.bc.ca) [46] In brief, these are extensive data resources for applied health services research with data holdings spanning information on all dispensed medications, includes drug name (brand and generic), Canadian Drug Identity Code, date dispensed, quantity dispensed, days of supply (BC PharmaNet), outpatient services, hospital separations, and vital statistics (Population Data BC).

### Outcomes

The primary outcome is adherence to ICS ascertained by the medication possession ratio (MPR), the ratio of the days of medication supplied over the one-year follow-up [47], which will be calculated using BC PharmaNet data. Secondary outcomes include: 1) asthma control at the end of follow-up (measured by the Asthma Control Test [41]; 2) asthma-related quality of life (measured by using the AQLQ [48]); 3) asthma-related hospital admissions; and 4) use of reliever medications during the follow-up period (ascertained from the administrative health data).

### Sample size calculation

Sample size calculations for cluster RCTs must take into account correlation among patients within clusters [49]. We used the formula for calculating sample size, *N*, for cluster RCTs for comparison of means by Donner and Klar [50] inflated by the design effect (inflation factor, 1 + (*m* – 1) *ρ*):
1

where *m* is the average cluster size and *ρ* is the coefficient of the intra-cluster correlation (ICC) or the ratio of between-cluster variance to total variance, σ^2^ [49], μ is the outcome (MPR), and Z indicates the normal Z-score. We drew from published ICC values based on adherence outcomes (0.0143 [51], 0.06 [52]), medication taking outcomes (0.00994 [53], 0.08 [54]), and non-adherence outcomes in cluster RCTs of adherence interventions (0.02 [55], 0.05 [56]). We also drew from our prior cluster RCT in community pharmacies for information on the number of clusters in that study (32 overall, 14 intervention, 18 usual care) [38]. Based on these data, we applied an ICC of 0.06 and calculated the number of patients that will be required to detect a 10% improvement in adherence rate - deemed clinically significant in prior studies [57] and also relevant from a cost-effectiveness perspective based on our previous research [58] - with a power of 80% and a significance level of 0.05, as 334. Accounting for approximately 10% attrition, we determined a target of 370 patients overall. The corresponding number of pharmacies (clusters) is 74 (37 randomized to intervention and 37 to usual care). As such, we target recruitment at five patients per pharmacy.

### Statistical analysis

#### General analytical framework

The analysis of the association of the intervention with the outcome will generally be based on regression analysis, and will be performed according to the intention-to-treat principle [59]. While randomization will in general cause balance in the distribution of covariates, regression-based adjustment for covariates further strengthen the inference [60]. To accommodate the nested structure of the data and variable follow-up times in a regression framework, generalized linear mixed models (GLMM) with appropriate distributions and link functions will be used. We will use the GLIMMIX procedure in SAS (version 9.3, SAS Institute Inc., Cary, North Carolina, United States) to fit such models. All significance levels will be based on two-tailed *P* values at 0.05, except for *post hoc* exploratory and secondary analyses, which will be adjusted for multiple-comparisons.

#### Covariates

All the analyses will be adjusted for the following variables: age, sex, baseline level of asthma control, socioeconomic status (income and education), and coverage by any third-party insurance. We will also draw on administrative health records from the 12-month period before the study entry to measure other potential confounders such as comorbid conditions and general pattern of healthcare utilization (number of physician visits, hospitalization, and medication dispensations).

#### Analysis for objectives

To evaluate the impact of the EmPhAsIS intervention on our primary outcome of one-year adherence as measured by the MPR, we will use a random effects GLMM, assuming an approximate normal distribution and specifying an identity link function. The estimation will be based on GLMM with a random-effects term for pharmacy and fixed-effects terms representing the intervention as well as covariates. The same analytical framework as above will be used for secondary outcomes. The choice of the distribution and link function will depend on the scale and type of the dependent variable. For asthma control, a random-effects ordinal logistic regression model (GLMM with binomial distribution and cumulative logit link function) will be performed. Normal distribution and identity link function (equivalent of a linear mixed model) will be used to predict longitudinal changes in AQLQ and ACT.

### Cost-effectiveness analysis

The ultimate figure of merit for the proposed intervention is whether the benefit of the program will justify the resources required for its implementation and operation. Once implemented, the intervention can provide longterm services; therefore, it is important to recognize the need for a rigorous economic evaluation of the program’s cost and effectiveness outcomes extrapolated beyond the time horizon of the RCT. Consequently, in line with other economic evaluations of asthma interventions [61], we chose a 10-year time horizon for this analysis. A probabilistic decision-analytic model will be used to perform a cost-effectiveness analysis from the societal perspective of the intervention compared with usual care. The key outcome of the cost-effectiveness analysis will be the incremental cost-effectiveness ratio (ICER), with quality-adjusted life-years (QALY) as the effectiveness measure. The ICER is defined as the difference in arithmetic mean costs between the intervention and usual care (*C*_*i*_ - *C*_*u*_) divided by the difference in arithmetic mean effectiveness (QALYs) between the same groups (*E*_*i*_ - *E*_*u*_).

**Figure 3:**
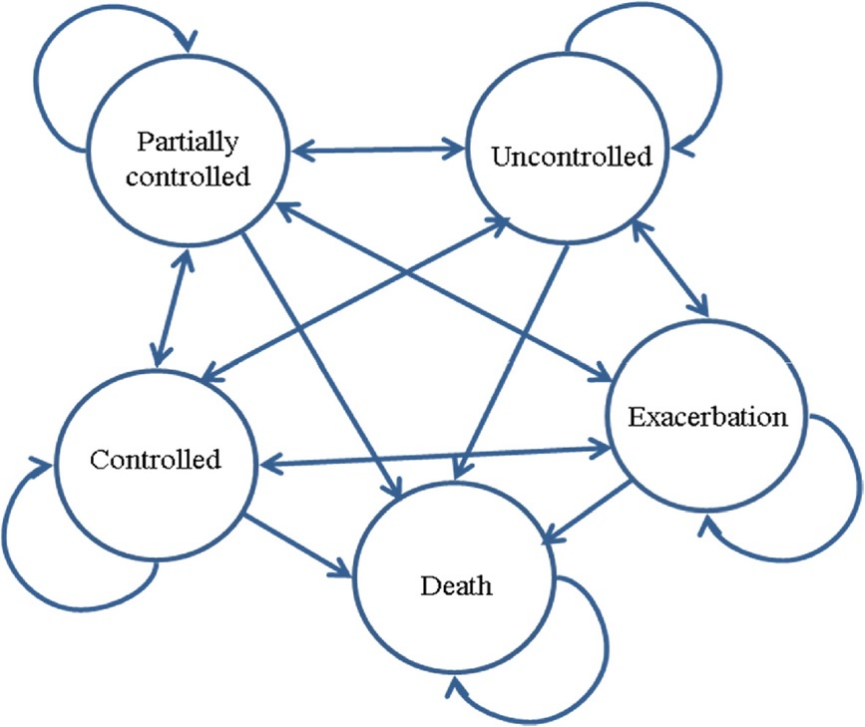
**Schematic illustration of the asthma Markov model.**

Co-investigators have already developed and calibrated a generic asthma Markov model that is capable of translating adherence at any value of MPR (the primary outcome) into transition rates across levels of asthma control and exacerbation health states, and eventually into costs and quality of life [62]. The core of the model is based on the concept of asthma control, as defined by GINA [6], with weekly transition cycles (Figure 3). We will update this model to a Canadian context and adapt it to incorporate specific aspects of the EmPhAsIS intervention. Two key study-specific components that will inform the analysis which will be estimated from the RCT data are the operating resource use of the intervention, and change in adherence due to the intervention. The cost of the intervention will be assessed from the society’s perspective by collecting the number of SMS transactions, phone interviews, and pharmacy visits. We will also ensure careful documentation of the time spent by the pharmacist delivering the intervention (phone calls and in-person visits). Protocol-driven resources (such as time spent to fill out the questionnaires) will be excluded from this analysis.

## Discussion

In a review of literature published between 1990 and 2002 of adherence interventions in asthma, Bender *et al*. summarized the three key shortcomings of previous adherence intervention studies: reliance on inadequate adherence measures, inclusion of convenience samples of well-motivated patients, and assessments of intervention outcomes artificially boosted by attrition of the least adherent [33]. The design of the EmPhAsIS trial overcomes these key shortcomings of the previous research. First, our objective assessment of adherence, made possible through the unique, data-rich environment of BC, protects us from the biases that arise when adherence is measured subjectively, and from the prohibitive costs and impracticalities of a direct measure of adherence, such as the Medication Event Monitoring System [63]. Second, by applying liberal inclusion criteria and a follow-up plan that does not disrupt individuals’ health behavior, our study is a pragmatic trial with a high degree of external validity. Third, by assessing the primary outcome variable (MPR) in a way that is not affected by voluntary withdrawal from the study, we protect our results from the attrition of the least adherent.

The merit of an mHealth technology is not solely a function of its clinical benefits. Large investments in mHealth may, by diverting resources, result in a shortfall in funding for basic infrastructure, equipment, and staffing elsewhere in the system [64]. Until mHealth interventions are ‘fit for purpose’, healthcare professionals are unlikely to adopt them and this risks implementation failure [64]. By focusing on a familiar and ubiquitous communication technology with low implementation and operation costs, and low burden on the clients (patients and pharmacists), we are confident of the successful uptake of our proposed intervention, provided that its merits are demonstrated.

Considering the prevalence of asthma, the extent of the non-adherence problem in this disease, and the availability of effective treatments, there is a tremendous potential to reduce the burden of asthma through improving adherence. This is the first study of an intervention based on mobile communication technology involving community pharmacists in asthma management. Our proposed intervention can also pave the way for the management of other chronic diseases through facilitating patients’ access to some of the most underutilized resources in the chain of healthcare delivery.

## Trial status

The EmPhAsIS trial is not yet recruiting. Recruitment is expected to begin January 2015.

## Authors’ information

Mary A De Vera and Mohsen Sadatsafavi are co-principal investigators.

## Abbreviations

AAAQ: Adult Asthma Adherence Questionnaire
ACT: Asthma Control Test
AQLQ: Asthma Quality of Life Questionnaire
BC: British Columbia
EmPhAsIS: Empowering pharmacists in asthma management through interactive SMS
GINA: Global Initiative for Asthma
GLM: Generalized linear models
ICC: Intra-class correlation
ICER: Incremental cost-effectiveness ratio
ICS: Inhaled corticosteroids
MPR: Medication possession ratio
MSP: Medical services plan
QALY: Quality-adjusted life-year
RCT: Randomized controlled trial
SMS: Short messaging service
UBC: University of British Columbia.
